# Supplementary material for: Promotion of Deoxycholic Acid Effect on Colonic Cancer Cell Lines In Vitro by Altering the Mucosal Microbiota
Source: Microorganisms. 2022 Dec 15;10(12):2486. doi: 10.3390/microorganisms10122486 (PMC9788287; doi:10.3390/microorganisms10122486)
Supplement: Supplementary file 1 [file microorganisms-10-02486-s001.zip › microorganisms-2052550-supplementary.pdf]

Supplementary Table S1. Primer sequences of real-time PCR

Gene Name	Froward primer (5′-3′)	Reverse primer (5′-3′)
18S rRNA	GATATGCTCATGTGGTGTTG	AATCTTCTTCAGTCGCTCCA
SHP	CCCCAAGGAATATGCCTGCC	TAGGGCGAAAGAAGAGGTCCC
FGF19	CCAGAAGACAGGCAGTAGT	CTGGAGGGA TTTGGGAAGG
FXR	AACCATACTCGCAATACAGCAA	ACAGCTCA TCCCCTTTGA TCC

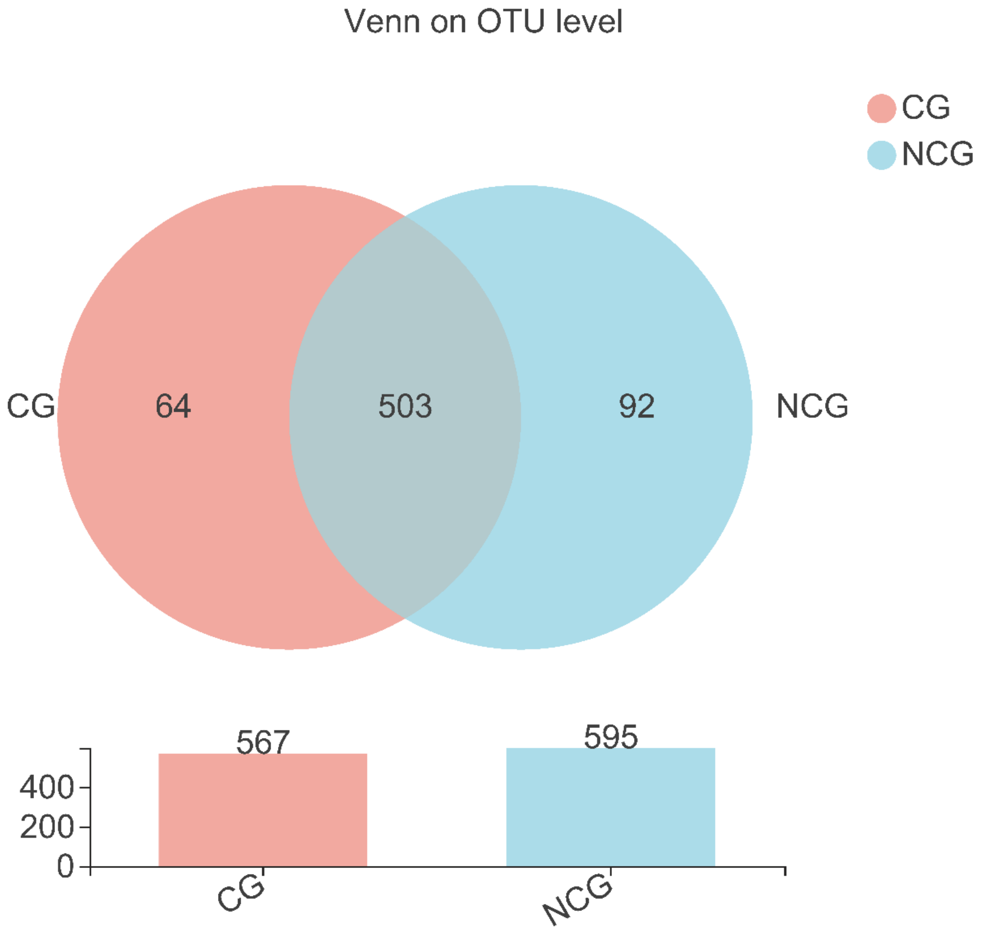

Supplementary Figure S1. The richness of bacterium using Venn figure of OTU level between cancerous group and non-cancerous group.

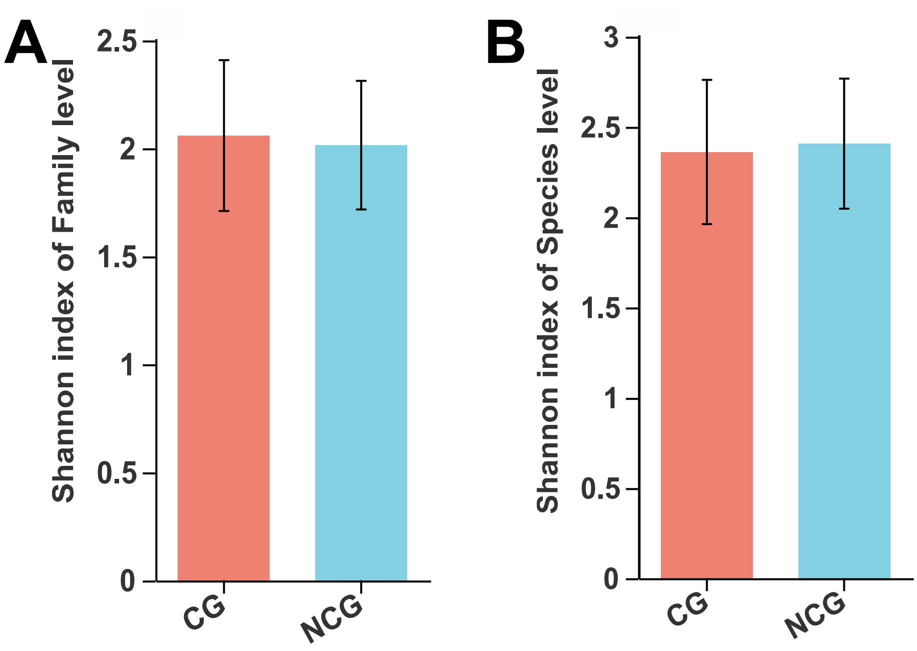

Supplementary Figure S2. The α diversity analysis using Shannon index of Family and Species level between cancerous group and non-cancerous group.
